# Characterisation of the *Vitis vinifera *PR10 multigene family

**DOI:** 10.1186/1471-2229-10-184

**Published:** 2010-08-20

**Authors:** Sylvain Lebel, Paul Schellenbaum, Bernard Walter, Pascale Maillot

**Affiliations:** 1 Université de Haute Alsace, Laboratoire Vigne, Biotechnologies & Environnement, 33 rue de Herrlisheim, BP 50568, 68 008, Colmar Cedex, France

## Abstract

**Background:**

Genes belonging to the *pathogenesis related 10 *(*PR10) *group have been studied in several plant species, where they form multigene families. Until now, such an analysis has not been performed in *Vitis vinifera*, although three different *PR10 *genes were found to be expressed under pathogen attack or abiotic stress, and during somatic embryogenesis induction. We used the complete genome sequence for characterising the whole *V. vinifera PR10 *gene family. The expression of candidate genes was studied in various non-treated tissues and following somatic embryogenesis induction by the auxin 2,4-D.

**Results:**

In addition to the three *V. vinifera PR10 *genes already described, namely *VvPR10.1*, *VvPR10.2 *and *VvPR10.3*, fourteen different *PR10 *related sequences were identified. Showing high similarity, they form a single cluster on the chromosome 5 comprising three pseudogenes. The expression of nine different genes was detected in various tissues. Although differentially expressed in non-treated plant organs, several genes were up-regulated in tissues treated with 2,4-D, as expected for *PR *genes.

**Conclusions:**

*PR10 *genes form a multigene family in *V. vinifera*, as found in birch, apple or peach. Seventeen closely related *PR10 *sequences are arranged in a tandem array on the chromosome 5, probably reflecting small-scale duplications during evolution. Various expression patterns were found for nine studied genes, highlighting functional diversification. A phylogenetic comparison of deduced proteins with PR10 proteins of other plants showed a characteristic low intraspecific variability. Particularly, a group of seven close tandem duplicates including *VvPR10.1*, *VvPR10.2 *and *VvPR10.3 *showed a very high similarity, suggesting concerted evolution or/and recent duplications.

## Background

PR10 proteins belong to the huge family of pathogenesis related (PR) proteins ubiquitous in the plant kingdom. PR proteins were first identified as defence molecules produced in response to pathogen attack and some of them actually display an antimicrobial activity. However, numerous studies have reported their induction under a great variety of abiotic stress conditions as well as possible constitutive or developmentally regulated expression [1]. Sharing common biochemical characteristics (acidic pI, resistance to proteolytic degradation, small molecular mass) PR proteins are divided into seventeen different groups based on their primary structure, serological relationships and biological activity [2]. Most of them are extracellular, but some others are found in the cytoplasm, mainly in the vacuole. PR10 proteins present the specificity to be free in the cytoplasm and are therefore classified as intracellular PR (IPR) proteins. They are closely related to a group of major tree pollen allergens and food allergens, that belong to the Bet v 1-like superfamily [3].

*PR10 *genes form multigene families with low intraspecific variation and higher interspecific variation that make them interesting phylogenetic markers [4-6]. Some of them were shown to be organized in chromosome clusters [7,8]. Characterised in a number of plant species, most *PR10 *genes share an open reading frame (ORF) from 456 to 489 bp interrupted by an intron of 76-359 bp at a highly conserved position [9]. This ORF codes for an acidic small protein with conserved sequence features: three amino acids E96, E148 and Y150 (as positioned in Bet v 1) possibly involved in ribonucleasic activity and two other remarkable domains comprising the motif GXGGXGXXK (aa 47-55) that forms a P-loop supposed to have NTPase activity and the Bet v 1 motif [PS00451] characteristic of proteins from the Bet v 1 superfamily [9].

The significance of multiple close copies of a gene in a single plant species has to be clarified. In birch or yellow lupine, some *PR10 *genes are constitutively expressed while others are induced under pathogen attack, abiotic stress or during plant development, suggesting functional diversification [10,11]. A significant common feature of PR10 proteins is a large Y-shaped hydrophobic cavity, as shown by the determination of three-dimensional structure [3,12-15]. This internal cavity could be responsible for the intracellular transport of apolar ligands, so diverse as fatty acids, flavonoids, cytokinins or brassinosteroids [10,14,16,17]. Slight modifications of the structure and shape of this cavity would allow to bind different ligands, what could account for the diverse roles hypothesised for PR10 proteins in plant defence and development [12,13].

To date, three different *PR10 *genes have been described in *Vitis vinifera*. *VvPR10.1 *was shown to be up-regulated during a pathogen interaction with *Pseudomonas syringae *in the cultivar Ugni blanc [18], while the expression of *VvPR10.1*, *VvPR10.2 *and *VvPR10.3 *was detected during somatic embryogenesis (SE) induction in the cultivar Chardonnay [19]. Moreover, several studies showed a strong and specific production of PR10 proteins in *V. vinifera *under salt or herbicide stress [20,21], as well as after fungal attack [22,23].

The recent publication of the whole genome sequence of *V. vinifera *by the Genoscope [24] and the Instituto Agrario San Michele all'Adige (IASMA) [25] makes genome scale analyses possible. Using these databases, we characterised all sequences of the *PR10 *gene family of *V. vinifera *and monitored the expression of nine candidate genes in various tissues and conditions.

## Results

### *In silico *identification of *V. vinifera PR10 *related sequences

**Figure 1:**
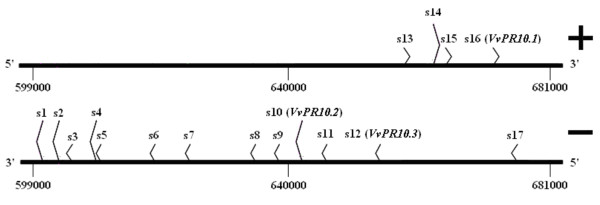
**Organisation of *V. vinifera PR10 *related sequences in a single cluster on chromosome 5**. All sequences are located between the positions 599,000 and 681,000, on the + or the - strand. Nucleotide positions are as referenced on the Genoscope website.

We searched for all *V. vinifera PR10 *related sequences in the whole genomic sequence published by the Genoscope. An automatic list of chromosome regions containing *PR10 *and *PR10-like *gene annotations was produced. After correction for mis- and redundant annotations, ten sequences with a complete ORF were retained, comprising *VvPR10.1 *and *VvPR10.2*, previously identified (AJ291705 and AJ291704 respectively) and the recently described *VvPR10.3 *gene (EU379313) [19]. Three additional incomplete sequences corresponded to pseudogenes. All thirteen sequences were arranged in a tandem array on the chromosome 5. On both sides of this cluster, we could identify four more *PR10 *related sequences, omitted by automatic annotation. No additional sequence was retrieved by a further homology search, suggesting that all *V. vinifera PR10 *genes are grouped in this single cluster, approximately 80,000 pb long and limited by putative genes not related to *PR10 *genes. The seventeen *PR10 *related sequences were named s1 to s17 and numbered according to their position on the chromosome regardless the strand they were located on (Figure 1): the twelve first (s1 to s12) and the last one (s17) on the minus (-) strand of the chromosome, and s13 to s16 on the plus (+) strand. *VvPR10.1*, *VvPR10.2 *and *VvPR10.3 *respectively correspond to s16, s10 and s12. All Genoscope references and precise locations are given in the additional file 1. The seventeen sequences collected from the Genoscope (PN 40024) were compared to their counterparts in the IASMA database (Pinot noir ENTAV 115), which are scattered on fifteen different scaffolds [additional file 2]. Each sequence was less than 2% divergent from its counterpart, highlighting a high similarity between the two genomes and databases.

### Characterisation and comparison of nucleotide sequences

**Figure 2:**
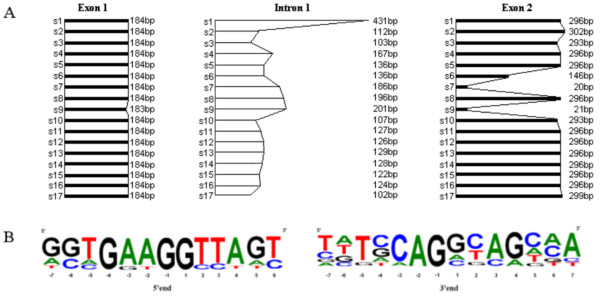
**Exon-intron structures of the seventeen *PR10 *related sequences**. A: length of exons (bold dashes) and introns (thin dashes). B: frequency of nucleotides at the 5' and 3' putative splicing sites of introns. The size of letters is proportional to the nucleotide frequency at each position. The numbers indicate nucleotide position relatively to the first nucleotide of intron 1 (number 1 at the 5' end) or to the first nucleotide of exon 2 (number 1 at the 3' end). *VvPR10.1*, *VvPR10.2 *and *VvPR10.3 *respectively correspond to s16, s10 and s12.

The putative exon-intron structure of the seventeen *PR10 *related sequences was determined (Figure 2a). Each sequence is interrupted by a single intron at a very well conserved position, presenting consensus motifs at the 5' and 3' boundaries with exons (Figure 2b). The hypothetical CDS are 204 to 486 bp long. The length of exons is well conserved, except for s6, s7 and s9, which are shortened by a premature stop codon suggesting that they are pseudogenes. In addition, s9 presents a single nucleotide deletion in the first exon, which induces a frame shift definitely compromising a correct translation. The structure of the fourteen sequences with a complete ORF is in accordance with previous reports on *Vitis vinifera *and other plant *PR10 *genes [9,18,19].

**Figure 3:**
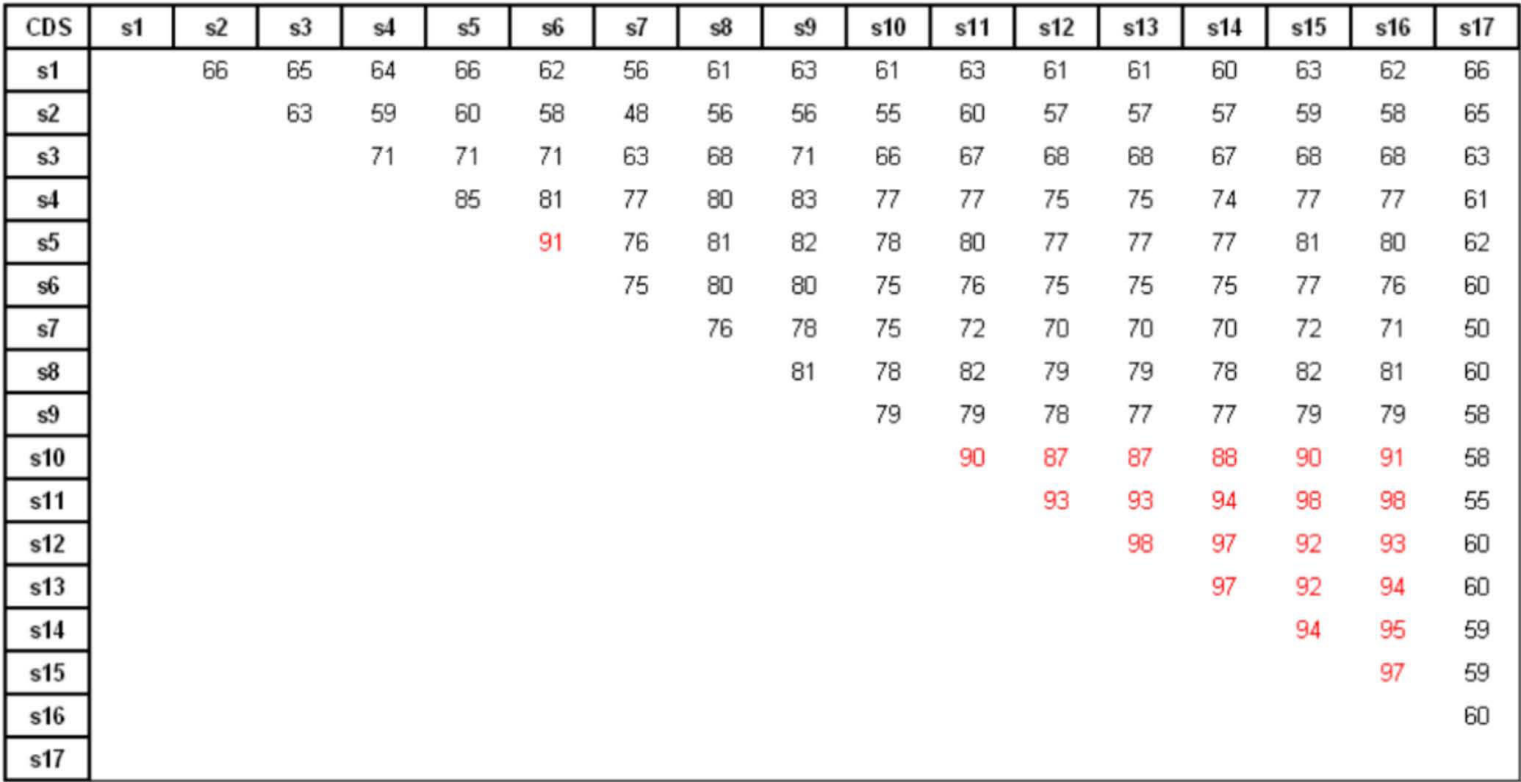
**Percentages of nucleotide similarity between the CDS of the seventeen sequences**. High percentages of nucleotide similarity are highlighted in red. *VvPR10.1*, *VvPR10.2 *and *VvPR10.3 *respectively correspond to s16, s10 and s12. The values were obtained from sequence alignments on ClustalW.

The CDS were aligned for comparison. The percentages of nucleotide similarity are given in Figure 3. All sequences are very similar and show at least 48% of nucleotide similarity. A very high similarity (≥92%) was found between the six sequences s11-s16, including *VvPR10.1 *and *VvPR10.3*, that are also strongly similar to s10-*VvPR10.2 *(87-91% of nucleotide similarity), together forming a group of closely related sequences. The sequence of the pseudogene s6 is also remarkably similar to s5 (91%). The intron sequence is more variable and the similarity percentages range from 8 to 100%, being very high for the seven sequences s10-s16 comprising *VvPR10.1*, *VvPR10.2 *and *VvPR10.3 *on one hand, and for s5 and s6 on the other hand [additional file 3].

### Prediction of expression

**Figure 4:**
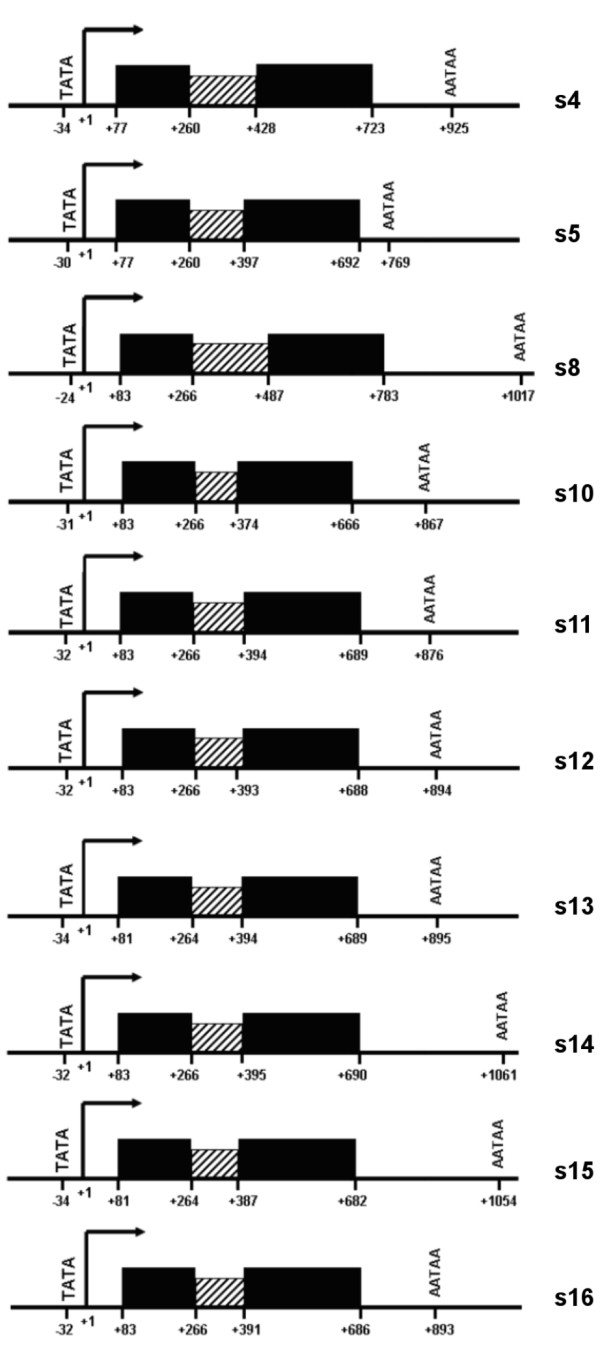
**Predicted structures for *PR10 *related sequences of *V. vinifera***. Only sequences having canonical transcription signals are shown. The arrow indicates the transcription starting site (+1). Predicted exons are represented as black boxes and deduced introns as dashed boxes. Given positions respectively correspond to the start of the TATA-box, the transcription starting site, the first nucleotide of the CDS, the last nucleotide of exon 1, the first nucleotide of exon 2, the last nucleotide of the CDS and the first nucleotide of the poly-A signal. Predictions were performed using the GeneFinding program. *VvPR10.1*, *VvPR10.2 *and *VvPR10.3 *respectively correspond to s16, s10 and s12.

In order to determine whether the newly identified *PR10 *related sequences could be functional genes, we searched for related Expressed Sequence Tags (ESTs) in the databases (Table 1). Consistent with the prediction of s6, s7 and s9 as pseudogenes, no EST was found to match these incomplete sequences. In addition, we found no EST corresponding to s2, s4, s8 nor s17, suggesting that these sequences would not be transcribed, at least in the tissues and conditions reported in the databases. On the contrary, numerous different ESTs (> 100) were found to match s11, s13, s14 and s15 with up to 99% nucleotide similarity. Although some of them also matched the highly homologous *VvPR10.1*, *VvPR10.2 *and *VvPR10.3*, the expression of which was reported [18,19], other ESTs were closer to s11, s13, s14 and s15, that would therefore be expressed in varied reproductive and/or vegetative tissues. Several ESTs corresponding to s1, s3 and s5 were also found (1, 52 and 4, respectively). Although a single incomplete EST (27% of coverage) matched s1, its sequence was 100% similar to the sequence of s1. Moreover, this EST covered the last 135 nucleotides of the CDS and 3'UTR, which are usually the most varying parts of a gene sequence, suggesting that this EST is a specific partial transcript of s1. The ESTs matching s3 and s5 with up to 99% similarity and 100% coverage argue for the expression of these two sequences. Using the Gene Finding program, a correct gene structure was predicted for the seven sequences s4, s5, s8, s11, s13, s14 and s15, in addition to *VvPR10.1*, *VvPR10.2 *and *VvPR10.3 *(Figure 4). All possess the well positioned TATA-box, transcription starting site (TSS) and polyadenylation signal, defining a CDS of 477-480 bp. No promoter was found for s1, s2, s3 and s17. When a manual search was performed, no TSS-motif was detectable for these sequences, but a TATA-box was identified for s1, s3 and s17 at the respective positions -1,214, -699 and -313 nucleotides. On the whole, in addition to the three well characterised *V. vinifera PR10 *genes, seven *PR10 *related sequences showed the canonical transcription signals, suggesting their possible expression, although no specific EST was found to match s4 nor s8. In contrast, s1 and s3 did not show the features usually described for effective transcription, but some specific ESTs were identified in the databases, indicating their probable expression. Finally, s2 and s17 showed no usual transcription signals, and no EST was found to match these two sequences.

**Table 1:** ESTs from *Vitis *spp. related to the seventeen *PR10 *sequences

Sequence	Number of ESTs	Best results		Best results found in
			
		Coverage %	Similarity %	
*VvPR10.1*	> 100	100	100	F, L, R, Be, Bu

*VvPR10.2*	> 100	100	100	F, L, R, Be, Bu

*VvPR10.3*	> 100	100	100	F, L, R, Be, Bu

s1	1	27	100	Be

s2	0			

s3	52	100	99	F, L, R, Be

s4	0			

s5	4	100	99	F, L, R, Be

s6	0			

s7	0			

s8	0			

s9	0			

s11	> 100	100	99	F, L, R, Be, Bu

s13	> 100	100	99	F, L, R, Be, Bu

s14	> 100	100	99	F, L, R, Be, Bu

s15	> 100	100	99	F, L, R, Be, Bu

s17	0			

BLAST results were obtained for the putative CDS of the seventeen sequences using the Vitis EST database of NCBI. The number of ESTs indicates the number of matching results for each query. The percentage of coverage indicates the relative length of coverage of the sequence by the best matching EST. The percentage of nucleotide similarity is given for the best matching EST. F = flowers; L = leaves; R = roots; Be = berries; Bu = buds.

### Characterisation of deduced PR10 proteins and phylogenetic analysis

**Figure 5:**
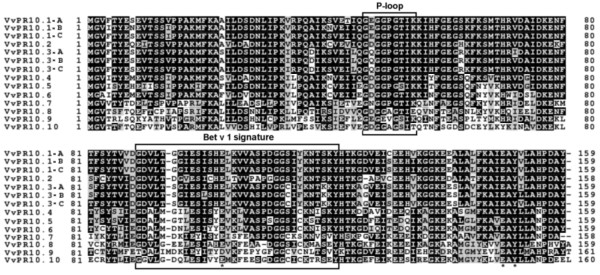
**Sequence alignment of deduced PR10 proteins**. The P-loop and Bet v 1 signature are framed. A star (*) marks the amino-acids implied in possible ribonucleasic activity (E102, E149 et Y151). Alignments were performed with ClustalW. A *V. vinifera *specific Bet v 1 motif was determined: G-[DG]-[VA]-L-x(4)-E-[SY]-[IL]-[CSATV]-[HY]-[ED]-x-[KST]-x-[VE]-x(3)-[GNDS]-G(2)-[CS]-x(2)-K-x(2)-[SK]-X-Y. In PR10.8 and PR10.10, the last aa varies in the P-loop motif (E and T, respectively); in PR10.9, E_102 _is replaced by D_102_, the 4^th ^aa in the P-loop motif is E, and the Bet v 1 motif presents 4 differences (out of 34 aa) at the positions 1, 6, 23 and 29; in PR10.6, there is one difference (out of 33 aa) in the Bet v1 motif (position 12).

**Figure 6:**
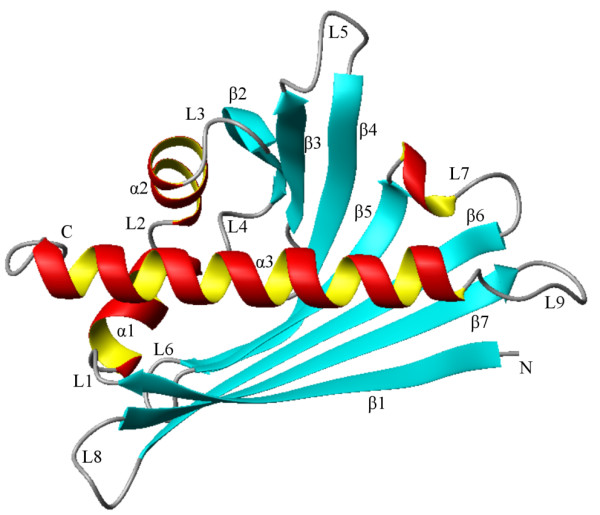
**Three-dimensional structure of *V. vinifera *PR10.1 represented by a ribbon diagram**. The structure was predicted on an automated comparative protein modeling server using SWISS-MODEL.

The nucleotide sequences with a complete ORF were named according to usual nomenclature. The highly similar sequences *VvPR10.1*, *s15 *and *s11 *on one hand, and *VvPR10.3*, s13 and s14 on the other hand (≥97% nucleotide similarity) were named *VvPR10.1-a*, *-b *and *-c*, and *VvPR10.3-a*, *-b *and *-c*, respectively. The other sequences were named *VvPR10.4 *to *VvPR10.10 *for s8, s5, s4, s3, s1, s2 and s17, respectively, following increasing divergence from the group of *VvPR10.1-a *to *VvPR10.3-c *sequences. Putative protein sequences were deduced from nucleotide sequences, analysed and compared. All proteins are 158-160 aa long (Figure 5), have a calculated molecular mass ranging from 17.1 to 18.4 kDa and a theoretical pI from 4.7 to 6.3, consistent with usual plant PR10 features [9]. Apart from few exceptions mentioned in the figure legend, all sequences present the three characteristic amino acids thought to be implied in ribonucleasic activity: E_102_, E_149 _and Y_151, _the characteristic P-loop motif (aa 47-55) described in various species and corresponding to the consensus sequence GXGGXGXXK, and the Bet v 1 motif which is the signature for the Pathogenesis-related proteins Bet v 1 family (Prosite accession PS00451)[3,9]. The predictive three-dimensional structure of the deduced proteins was determined. As for PR10.1 (Figure 6), all proteins would be composed of three alpha-helices and a seven-stranded antiparallel beta-sheet arranged to form a large internal hydrophobic cavity. Nine short loops connect the secondary elements. This conformation is in accordance with the crystal structure determined for plant PR10 proteins in cherry, yellow lupine, birch and celery [12-15]. All these observations suggest correct structure and folding for the eleven putative *V. vinifera *PR10 proteins in addition to PR10.1, PR10.2 and PR10.3 [additional file 4].

**Figure 7:**
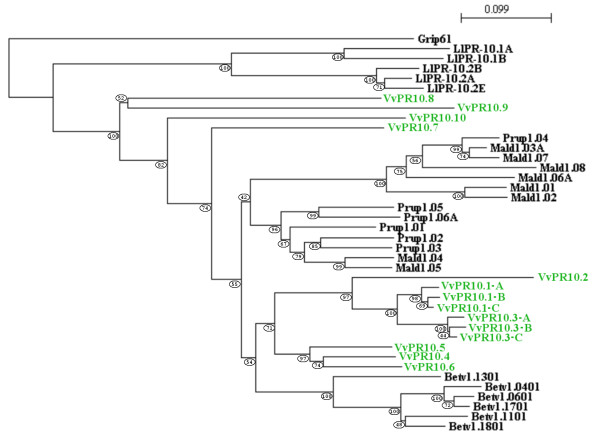
**Phylogenetic relationships between *V. vinifera *PR10 proteins and representative PR10 proteins from *Betula pendula*, *Lupinus luteus*, *Malus domestica *and *Prunus persica***. GenBank accession numbers are as follows: *Betula pendula *Betv1.0401 (CAA54482), Betv1.0601 (CAA54484), Betv1.1101 (CAA54694), Betv1.1301 (CAA54696), Betv1.1701 (CAA96539) and Betv1.1801 (CAA96540); *Lupinus luteus *LlPR10.1A (CAA56298), LlPR10.1B (CAA56299), LlPR10.2A (AAF77633), LlPR10.2B (AAF77634) and LlPR10.2E (AAP37978); *Malus domestica *Mald1.01 (AAX18288), Mald1.02 (AAX18291), Mald1.03A (AAX18313), Mald1.04 (AAX18294), Mald1.05 (AAX18296), Mald1.06A (AAX18299), Mald1.07 (AAX18307) and Mald1.08 (AAX18310); *Prunus persica *Prup1.01 (ACE80940), Prup1.02 (ACE80942), Prup1.03 (ACE80944), Prup1.04 (ACE80946), Prup1.05 (ACE80948) and Prup1.06A (ACE80952). The *V. vinifera *Grip61 gene (CAB85634) was included as an outgroup representative of another Bet v 1 subfamily [3]. The NJ-tree was generated with the Phylo_win program. The bootstrap value is given for each node.

A phylogenetic tree shows the relationships between *V. vinifera *PR10 proteins and representatives of the main PR10 subfamilies in other plants: Mal d 1 and Pru p 1 subfamilies I-IV in *Malus domestica *[7] and *Prunus persica *[8], Bet v 1 subfamilies I-III and V in *Betula pendula *[26] and LlPR10 subclasses I and II in *Lupinus luteus *[11] (Figure 7). In each species, PR10 proteins tend to form one (*Betula pendula, Lupinus luteus*) or two clades *(Malus domestica, Prunus persica *and *Vitis vinifera*), showing a low intraspecific and a higher interspecific variation, as previously described [4-6]. In a first clade, *V. vinifera *PR10 proteins are subdivided into two distinct homogeneous groups: PR10.1-A to PR10.3-C with more than 79% of amino acid identity and PR10.4 to PR10.6 with more than 81% of amino acid identity. These two groups of tightly phylogenetically connected proteins are closer to birch Bet v 1 than to PR10 proteins of other plant species, and are undoubtedly distant from the four other *V. vinifera *PR10 proteins, PR10.7 to PR10-10.

### Expression of *V. vinifera *candidate *PR10 *genes

The possible expression of ten out of the fourteen complete *PR10 *sequences was assessed in various non-treated tissues of *V. vinifera *cv. Chardonnay, as well as during secondary somatic embryogenesis. It was not possible to design specific primers for the detection of *VvPR10.1-b *and *-c *and *VvPR10.3-b *and *-c *transcripts, because of very high similarity with *VvPR10.1-a *and *VvPR10.3-a *transcripts respectively, even in the 5' and 3' UTRs.

**Figure 8:**
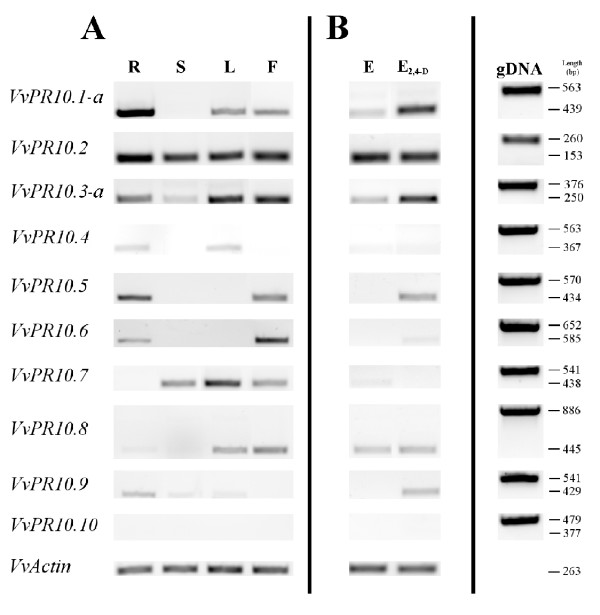
**Expression of *V. vinifera PR10 *genes**. A: tissue-specific expression; R = roots, S = stems, L = leaves, F = flowers. B: expression during secondary somatic embryogenesis induction; E = non-treated somatic embryos at the cotyledonary stage, E_2,4-D _= calli obtained from embryos treated with 2,4-D. gDNA = genomic DNA. The length of amplified sequences are given in bp.

Nine different genes were expressed in the tissues analysed, *VvPR10.10 *transcripts being undetectable in any condition (Figure 8). Moreover, *VvPR10.4 *and *VvPR10.9 *transcripts were only weakly detected in a few tissues.

In intact tissues, all nine *PR10 *genes were expressed, depending on the plant organ. In roots, the expression of all genes except *VvPR10.7 *was detected, although *VvPR10.8 *transcription was very weak. At the opposite, *VvPR10*.7 transcription was high in leaves, while *VvPR10.5 *and *VvPR10.6 *transcripts were not detected; all other genes were expressed at varied levels. In stems, *VvPR10.2*, *VvPR10.3-a *and *VvPR10.7 *were the only clearly expressed genes. Immature flowers expressed all genes except *VvPR10.4 *and *VvPR10.9*. In non-treated somatic embryos, *VvPR10.1-a, VvPR10.3-a *and *VvPR10.8 *transcripts were weakly detected, while *VvPR10.2 *transcription was clear.

The 2,4-D treatment of embryos used for induction of secondary somatic embryogenesis increased the expression of *VvPR10.1-a*, *VvPR10.3-a*, *VvPR10.5 *and *VvPR10.9 *but not *VvPR10.2 *and *VvPR10.8*. The expression of *VvPR10.6 *was also weakly stimulated. On the other hand, *VvPR10.7 *transcription was weakly detectable in intact embryos but not at all in tissues derived from 2,4-D-treated embryos. *VvPR10.4 *expression was not detected in non-treated or treated embryos.

## Discussion

We found a total of seventeen *PR10 *related sequences in the whole *V. vinifera *genome. Thirteen unique sequences were retained from an automatic search that initially produced ninety regions, reflecting redundancy of the database as well as annotation errors partly due to wrong homology detection. A manual search allowed the recovery of four additional *PR10 *related sequences. All seventeen sequences were found in a single compact cluster on the chromosome 5. Plant *PR10 *belong to multigene families. There are at least five *PR10 *genes in pea [27], eighteen *Mal d 1 *genes in apple [7], ten *Bet v 1 *genes in birch [6], eight *Fra a 1 *genes in strawberry [28], six *PR10 *genes in *Solanum surattense *[29], eight in yellow lupine [11], five in rice [30], and eight *Pru p 1 *and *Pru d 1 *genes in peach and almond, respectively [8]. They were shown to form physical clusters in apple [7] and peach [8]. Poplar *PR10 *genes are also supposed to be grouped on chromosomes [26]. Tandem duplicates are frequent in plant genomes and represent up to 16% of *Arabidopsis *genes [31]. Such gene clusters are thought to be produced by successive single gene or small-scale duplications. We found that thirteen out of the seventeen *V. vinifera PR10 *sequences are present on the chromosome in direct orientation, suggesting that most copies were produced by unequal crossing over events, as described in *Arabidopsis *and rice [31].

Following duplication, new copies of a gene may undergo modifications allowing functional diversification, which is a significant source of evolutionary novelty in plants [32]. However, gene duplication mostly creates copies that are rapidly lost through pseudogenisation. As a result, from numerous homologous sequences coexisting in a genome, only a part are functional genes. From the seventeen *V. vinifera PR10 *sequences, only fourteen have an intact ORF. In birch, the copy number of *PR10 *genes varies from twelve to twenty-five, depending on the species, and pseudogenes represents as much as one-third of *Betula nigra *alleles [6]. In *V. vinifera*, the pseudogenes s7 and s9 share the highest nucleotide similarity with *VvPR10.6 *(s4), suggesting that they could derive from its duplication. Likewise, s6 is closer to *VvPR10.5 *(s5) than to all other sequences and could therefore originate from its duplication. Moreover, s6 and *VvPR10.5 *are also very similar at the intron level (81% of nucleotide similarity), indicative of possible recent duplication or slow pseudogenisation.

Apart from pseudogenes, sequence divergence is generally reduced in *PR10 *multigene families. *PR10 *genes within a species are generally very similar and more distant from gene copies of other plant species [4]. Such a low intraspecific variability has been observed in *Passiflora *[5] and in the *Betula *genus [6]. The different paralogs are thought to undergo strong concerted evolution which is the tendency of a family of repeated genes to jointly evolve. The close physical proximity of tandem duplicates facilitates gene conversion or unequal crossing-over events leading to concerted evolution [6]. The phylogenetic tree in Figure 7 illustrates probable concerted evolution in the *V. vinifera PR10 *family. However, *V. vinifera *PR10 proteins divide into two clades interrupted by PR10 of other plants, indicating partial independent evolution. Interestingly, the seven sequences s10 to s16 corresponding to the very homologous proteins PR10.1-A to PR10.3-C follow one another on the chromosome, what could favor concerted evolution and suggest strong local selection pressure. However, it cannot be ruled out that recent duplication events produced these seven very homologous sequences.

Grapes, as other eudicot species, probably originate from an ancient hexaploid ancestor formed through whole genome duplication (WGD) events [24,33]. Recurrent polyploidisation is tightly linked to evolution in angiosperms, providing raw materials for gene diversification and genome refinement, and coinciding with species radiation [32]. A WGD is followed by incomplete and asymmetric loss of gene copies and chromosome rearrangements allowing the recovery of a diploid-like state compatible with effective reproduction. In *Arabidopsis*, it was possible to track gene pairs released by a recent specific WGD event [34]. Only 28.6% of gene pairs were retained from the transient tetraploid genome, the other pairs having lost a copy from one of the homeologous chromosomes. In *V. vinifera*, *PR10 *sequences were solely located on the chromosome 5, suggesting loss of some of the triplicate ancestral copies and/or translocation on a unique chromosome. The seventeen present *PR10 *sequences are most probably due to repetitive small duplications having continuously occurred during *V. vinifera *evolution. Subsequent mutations and possible positive selection are responsible for the observed divergence within the sequences. However, at least a part of *V. vinifera PR10 *sequences are probably subjected to concerted evolution, reducing variability and hampering the identification of putative triplicate ancestral copies.

We were able to detect the expression of nine out of the fourteen complete *PR10 *related sequences in varied non-treated and treated tissues of *V. vinifera *cv. Chardonnay. Three genes, *i.e*. *VvPR10.1-a*, *VvPR10.2 *and *VvPR10.3-a*, were already shown to be expressed under pathogen attack or abiotic stress, and during somatic embryogenesis induction [18,19], whereas expression of *VvPR10.4-VvPR10.9 *was never studied before. *VvPR10.10 *transcripts were not detectable in our conditions. This sequence has no corresponding EST in the databases and lacks canonical transcription signals. However, expression could be limited to very specific tissues and/or developmental stages. Although specific ESTs in the databases suggest a probable expression of the four sequences *VvPR10.1-b *and -*c *and *VvPR10.3-b *and *-*c, the distinction of each transcription product would require more sensitive methods than PCR, such as cDNA-AFLP or SSCP, which were used for discriminate highly homologous sequences in potato, polyploid cotton and barley [35-37]. The expression of *VvPR10.5*, *VvPR10.7 *and *VvPR10.8 *is not surprising because well matching ESTs were found in the databases. On the contrary, no EST was found to correspond to *VvPR10.4*, *VvPR10.6 *and *VvPR10.9*, whose expression is therefore reported for the first time. Interestingly, *VvPR10.7*, *VvPR10.8 *and *VvPR10.9 *are expressed although devoid of canonical transcription signals.

We found transcripts of all nine *PR10 *genes in varying amounts, in the different non-treated tissues analysed, suggesting a role apart from defence. Specific expression profiles were found in intact tissues, except for *VvPR10.2 *and *VvPR10.3-a*. Stems and intact embryos expressed a reduced subset of *PR10 *proteins. On the contrary, immature flowers expressed a large subset of *PR10 *genes, suggesting a possible role of some PR10 proteins during sexual reproduction. No *V. vinifera PR10 *gene was solely expressed in calli derived from 2,4-D-treated embryos. However, expression of several genes was enhanced in these tissues, at least weakly. Somatic embryogenesis is generally obtained from tissues subjected to the auxin 2,4-D, which acts as a stress factor able to trigger the reprogramming of plant somatic cells towards embryogenesis [38]. As a consequence, high amounts of defence proteins are produced following a 2,4-D treatment, as shown in grapevine cultures [39]. Consistent with the results reported here, we previously showed that varied *PR *genes including *VvPR10.1-a *and *VvPR10.3-a *are up-regulated during secondary somatic embryogenesis induction in *V. vinifera *[19]. *VvPR10.1 *expression was also previously reported to be induced in whole plant leaves challenged with the incompatible bacterium *Pseudomonas syringae *[18]. Interestingly, *VvPR10.4 *and *VvPR10.7 *were not responsive to 2,4-D treatment.

On the whole, various expression patterns were found for *V. vinifera PR10 *genes, indicating functional diversification and possible tissue specificity. Likewise, in other plants, PR10 proteins show expression diversity suggesting various biological activities [9]. A major feature of PR10 proteins is their internal hydrophobic cavity with openings at the protein surface. Several three-dimensional modeling studies showed that this structure is suitable for the binding and transport of diverse hydrophobic ligands as brassinolides [14] or homocastasterone [12]. Some birch and yellow lupine PR10 can bind diverse molecules such as cytokinins, brassinosteroids, fatty acids and flavonoids, suggesting that they could interfere with the trafficking of hormones inside the cell [10,16,17]. Moreover, overexpression of a *PR10 *gene in pea led to a change in the ratio between cytokinins and abscisic acid, showing that the PR10 content could be relevant for intracellular hormone regulation [40]. Although all crystallographic models for PR10 proteins share the same canonical fold, their superimposition can reveal structural differences [12]. Remarkably, the volume of the internal cavity and of its openings can show some variability, what could influence the type of transported ligand. In yellow lupine, different shapes for the inducible LlPR10.1A and the constitutive LlPR10.1B could account for their different biological roles [13].

## Conclusion

The availability of the complete genome sequence of *V. vinifera *allowed us to characterise the *PR10 *gene family. Seventeen different *PR10 *related sequences including three pseudogenes were identified and located in a single compact cluster on the chromosome 5, most probably reflecting repetitive small duplications during evolution. A phylogenetic analysis showed a characteristic low variability within the different sequences, especially within seven sequences closely located on the chromosome, suggesting probable concerted evolution. We could analyse the expression of nine genes in various tissues. Different expression patterns indicate functional specialisation. Several genes showed a typical stress induced up-regulation. Further experiments will help to elucidate the differential regulation of *V. vinifera PR10 *gene expression.

## Methods

### *In silico *identification of *PR10 *related sequences in the *V. vinifera *genome and characterization of candidate genes

The grapevine Genoscope database was used to identify any sequence related to *PR10 *genes [41]. The Genoscope Blat tool [42] and the ClustalW alignment tool of the European Bioinformatics Institute (EBI) [43] were used to retrieve and compare the sequences. An automatic list produced twenty-three unique (among ninety) putative *PR10 *or *PR10-like *gene annotations. Seven annotations with no consistent homology with *PR10 *genes were removed. Six other annotations covered three complete sequences, from which each exon was annotated as an independent gene, suggesting that the intron splicing sites were not detected. The last ten annotations comprised seven sequences with a complete ORF, and three with a prematurely interrupted ORF. All thirteen *PR10 *related sequences were found to form a cluster on the chromosome 5, between the positions 604,886 and 675,550. Thoroughly examining this chromosome portion as well as its 5' and 3' extensions, we found four additional annotations with nucleotide similarity (48-71% in the ORF) to the thirteen previous sequences. The *PR10 *cluster was found to be limited by two putative genes not related to *PR10 *sequences GSVIVT00033064001 and GSVIVT00033091001.

All corresponding genome sequences originating from the Istituto Agrario San Michele all'Adige (IASMA) grapevine database were retrieved using the NCBI Blast tool [44]. They were compared to the Genoscope sequences using the ClustalW alignment tool.

The exon-intron composition of *PR10 *sequences was automatically determined by the Gaze program on the Genoscope website. When necessary, the position of the intron was corrected relatively to the structure of *VvPR10.1 *[18], and according to the 5' and 3' splicing consensus sequences NN/GT and AG/NN, respectively.

*Vitis sp*. ESTs were searched, using the blastn program of the NCBI database [44].

Gene structures were predicted using the Gene Finding program on the University of London bioinformatics web server [45]. The organism parameter was set on "dicots". The FGENESH method showed the position of the TATA-box, the first and last nucleotides of the exons and the position of the polyadenylation signal. The TSSP-TCM method localized the transcription initiation site (+1).

### Protein sequence analysis

The putative CDS were translated using the Transeq program [46]. The PM and pI were determined using the COMPUTE program [47]. The PROSITE database [48] was used to find the Pathogenesis-related proteins Bet v 1 family signature. Three-dimensional structures were predicted on an automated comparative protein modeling server using SWISS-MODEL [49].

### Phylogenetic analysis and multiple alignments

A phylogenetic tree was built using the Neighbor-Joining method, with the Phylo_win program [50]. Bootstrap values were obtained from 500 replicates. The ClustalW alignment tool was used to compare protein sequences.

### Plant material

All expression studies were performed on *V. vinifera *plants or cultures of the cultivar Chardonnay.

We separately collected roots, stems (with nodes) and whole leaves from well developed plantlets (10-12 leaves) obtained from *in vitro *microcutting, as previously described [51].

Immature flowers were harvested from French vineyards, at the stage of separate flower buds.

Recurrent *in vitro *embryogenesis was induced from nodal explants of the *V. vinifera *cv. Chardonnay, as previously described [19]. Young cotyledonary somatic embryos were isolated and transferred onto a medium containing the auxin analog 2,4-D for induction of indirect secondary embryogenesis. After three weeks, an undifferentiated callus was obtained from each embryo.

Tissues were collected, frozen in liquid nitrogen and further conserved at -80°C until RNA extraction. Two independent samplings were performed.

### RNA extraction and analysis

Total RNA extractions and semi-quantitative RT-PCR experiments were performed as reported [19]. Primer pairs were designed to overlap the intron, in order to distinguish possible genome DNA contamination from gene expression products (Table 2). All primer pairs were shown to efficiently detect the respective corresponding genomic sequences (gDNA). Semi-quantitative RT-PCR tests were performed in three replicates. PCR products were sequenced to confirm their identity (Genoscreen, France).

**Table 2:** Sequences of primers used for semi-quantitative RT-PCR

Gene	GenBank	Forward primer 5'→3'	Reverse primer 5'→ 3'
*Actin*	AF369525	TGCTATCCTTCGTCTTGACCTTG	GGACTTCTGGACAACGGAATCTC

*VvPR10.1*	AJ291705	GAAATCATACAAGGAGAGGGAGGC	GCCAAACTTATTGAGACTGATAGGTG

*VvPR10.2*	AJ291704	CGATCACAGTGTAGCGGAATGAGAAT	AAGCTATCAAGTGCGTGGAAGTCATT

*VvPR10.3*	EU379313	GAAATCCTACAAGGACAGGGAGGT	CGGCCTTGGTGTGGTACTTTT

*VvPR10.4*	-	ATCCTTCCCCAAGCTATCAAG	GATTTGCCAAGAGGTAAGCC

*VvPR10.5*	-	ATCCTTGACTCTGATAACCTCA	ATGATATGAGACAAAGGAGTTTC

*VvPR10.6*	-	GTCCTTGATGTTGATAACCTC	AAGCCAAGCCTTTTAACTG

*VvPR10.7*	-	ATCGTCCCTCAGGCCATTA	AAGTGATTAAGTGGAGGAGAAGC

*VvPR10.8*	-	CTCTTGCCCCAGACCATAAG	ACATTGGACAACAGAGAAGTGAC

*VvPR10.9*	-	CAGTCAAAAGTACGCGACTCA	AAGTATAGGCGCGAGGGTGT

*VvPR10.10*	-	AATCAGTAAAGAGCATCGAGTT	AAAGTAATCACAACTCCTCGTC

## Authors' contributions

SL performed all *in silico *analyses as well as RT-PCR experiments. PS participated in studies performed *in silico*. PM carried out the tissue cultures. PS, BW and PM designed and coordinated the study. SL and PM wrote the manuscript. All authors read and approved the manuscript.

## Supplementary Material

Additional file 1 **Genome position and annotation reference for *PR10 *related sequences, as given on the Genoscope website**. (*) a double annotation means that the sequence has been annotated as two individual genes. Click here for file

Additional file 2 **Concordance of the map of the *PR10 *cluster obtained from the Genoscope and homologous scaffolds originating from the IASMA genome database**. Each coloured band corresponds to a different scaffold, given with GenBank accession number. IASMA sequences were obtained from NCBI. Click here for file

Additional file 3 **Percentage of nucleotide similarity between the introns of the seventeen sequences**. High percentages of nucleotide similarity are highlighted in red. *VvPR10.1*, *VvPR10.2 *and *VvPR10.3 *respectively correspond to s16, s10 and s12. The values were obtained from sequence alignments on ClustalW. Click here for file

Additional file 4 **Three-dimensional structure of deduced *V. vinifera *PR10 proteins represented by a ribbon diagram**. The structure was predicted on an automated comparative protein modeling server using SWISS-MODEL. With reference to PR10.1, PR10.8 and PR10.9 have a longer C-terminal end, while PR10.7 and PR10.10 have a shorter C-terminal end. The folding of the regions between α2 and β4 diverges from the model in PR10.5, PR10.6 and PR10.7. Click here for file
