# Supplementary figures and images for: Characterisation of the Vitis vinifera PR10 multigene family
Source: BMC Plant Biol. 2010 Aug 20;10:184. doi: 10.1186/1471-2229-10-184 (PMC3095314; doi:10.1186/1471-2229-10-184)

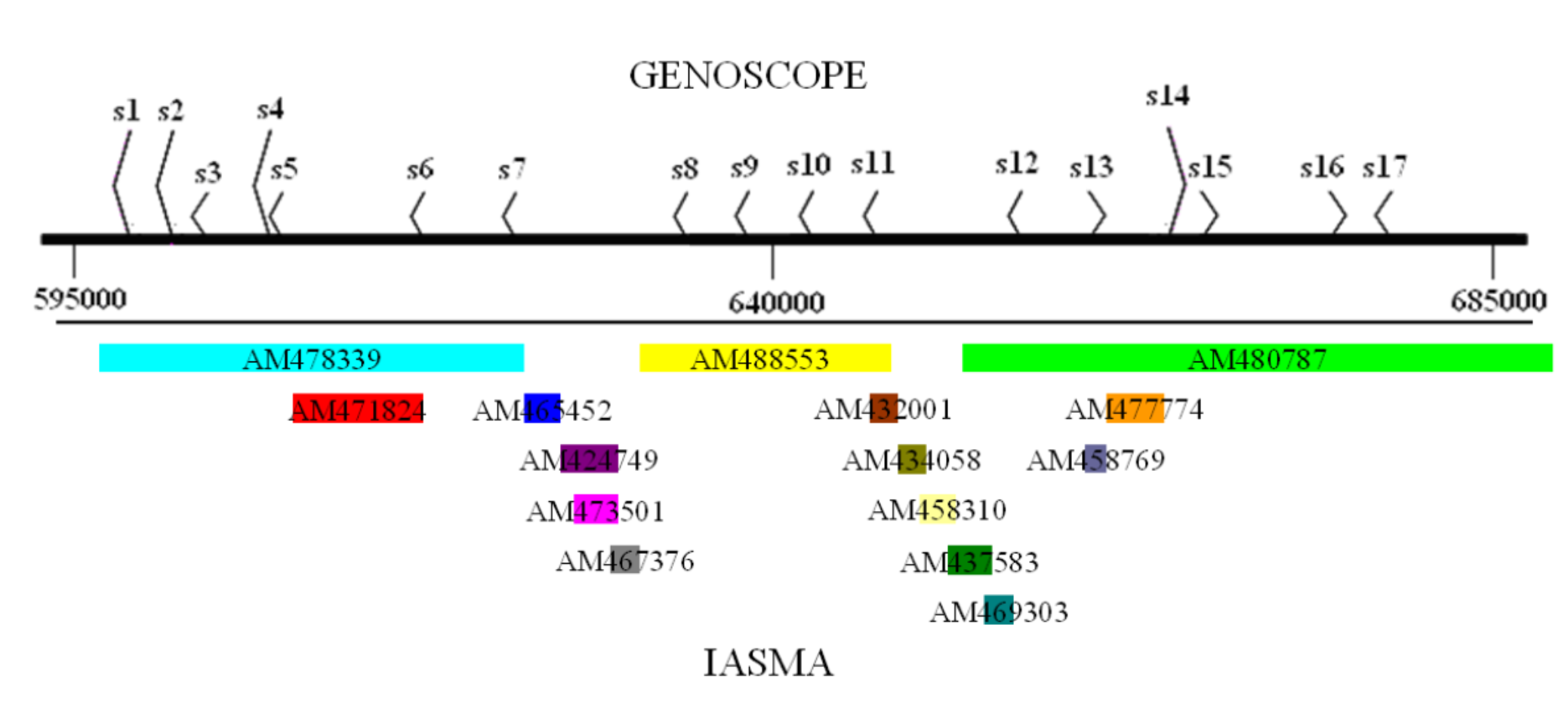

Supplement: Additional file 2 — Concordance of the map of the PR10 cluster obtained from the Genoscope and homologous scaffolds originating from the IASMA genome database. Each coloured band corresponds to a different scaffold, given with GenBank accession number. IASMA sequences were obtained from NCBI. [file 1471-2229-10-184-S2.TIFF]

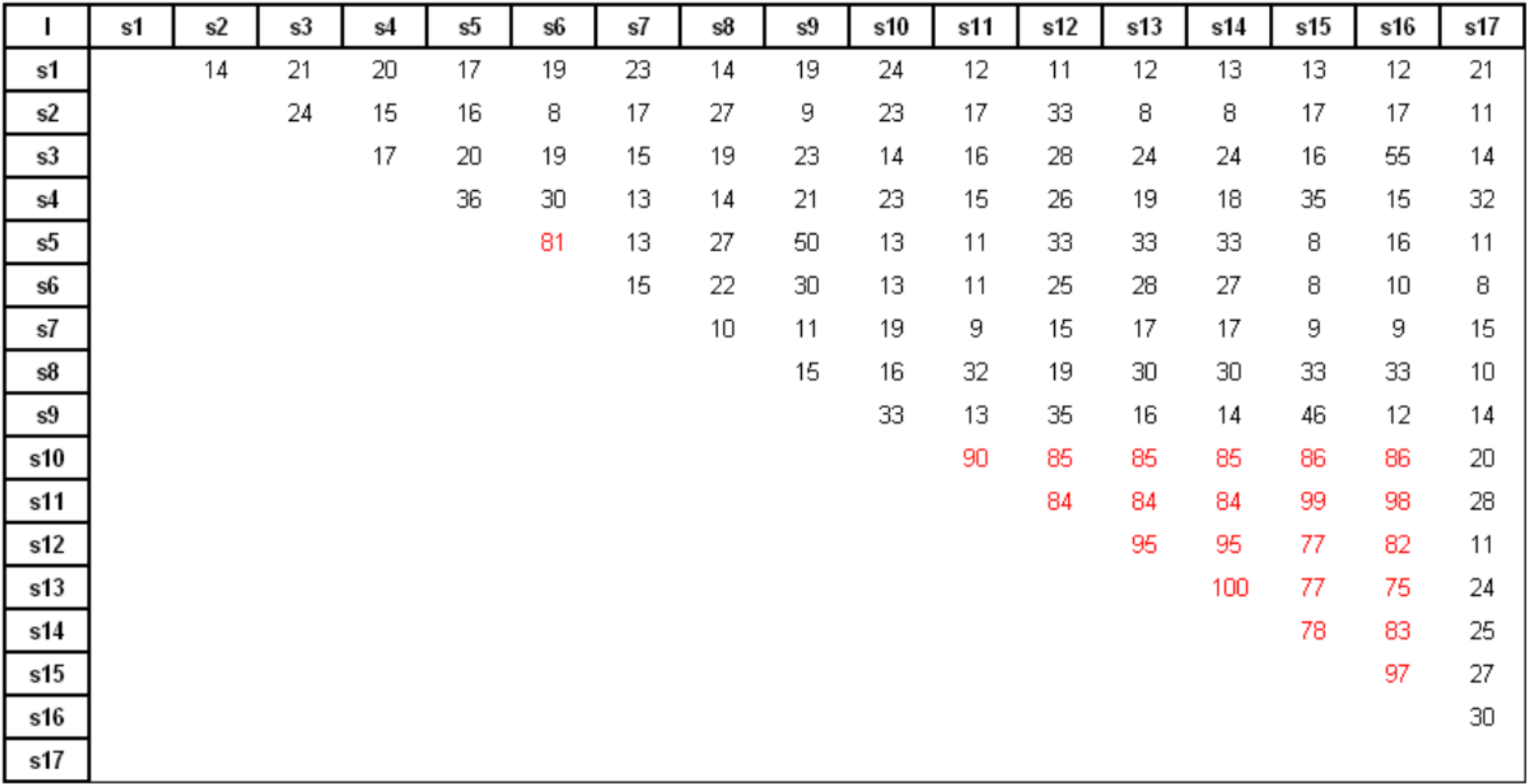

Supplement: Additional file 3 — Percentage of nucleotide similarity between the introns of the seventeen sequences. High percentages of nucleotide similarity are highlighted in red. VvPR10.1, VvPR10.2 and VvPR10.3 respectively correspond to s16, s10 and s12. The values were obtained from sequence alignments on ClustalW. [file 1471-2229-10-184-S3.TIFF]

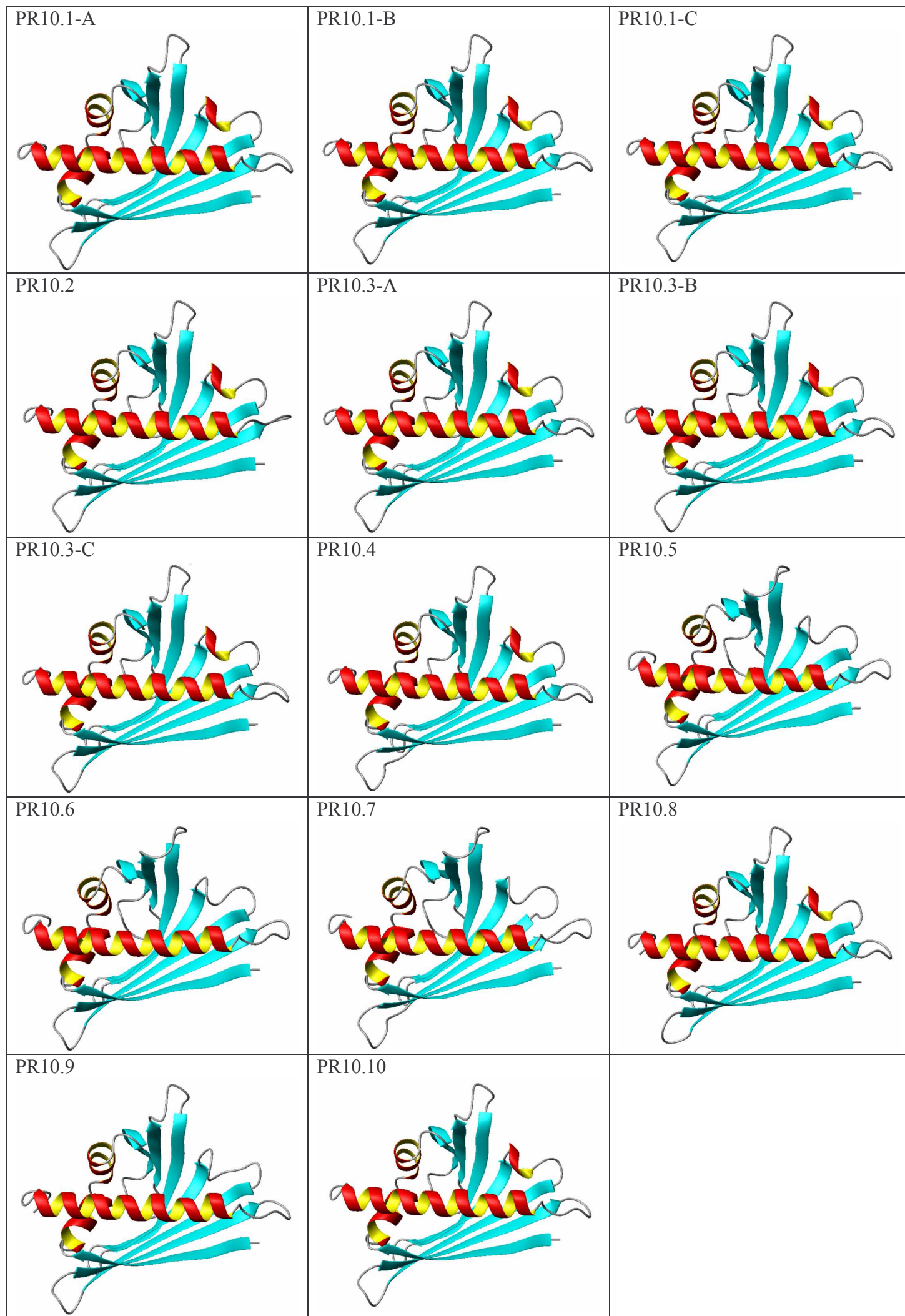

Supplement: Additional file 4 — Three-dimensional structure of deduced V. vinifera PR10 proteins represented by a ribbon diagram. The structure was predicted on an automated comparative protein modeling server using SWISS-MODEL. With reference to PR10.1, PR10.8 and PR10.9 have a longer C-terminal end, while PR10.7 and PR10.10 have a shorter C-terminal end. The folding of the regions between α2 and β4 diverges from the model in PR10.5, PR10.6 and PR10.7. [file 1471-2229-10-184-S4.PDF]
